# Glycan microarray reveal induced IgGs repertoire shift against a dietary carbohydrate in response to rabbit anti-human thymocyte therapy

**DOI:** 10.18632/oncotarget.23096

**Published:** 2017-12-11

**Authors:** Ron Amon, Shani Leviatan Ben-Arye, Limor Engler, Hai Yu, Noha Lim, Ludmilla Le Berre, Kristina M. Harris, Mario R. Ehlers, Stephen E. Gitelman, Xi Chen, Jean-Paul Soulillou, Vered Padler-Karavani

**Affiliations:** ^1^ Department of Cell Research and Immunology, Tel Aviv University, Tel Aviv, Israel; ^2^ Department of Chemistry, University of California-Davis, Davis, CA, USA; ^3^ Biomarker Discovery Research, Immune Tolerance Network, Bethesda, MD, USA; ^4^ Centre de Recherche en Transplantation et Immunologie UMR 1064, INSERM, Université de Nantes, Nantes, France; ^5^ Institut de Transplantation Urologie Néphrologie (ITUN), CHU Nantes, Nantes, France; ^6^ Clinical Trials Group, Immune Tolerance Network, San Francisco, CA, USA; ^7^ Division of Pediatric Endocrinology and Diabetes, University of California San Francisco, San Francisco, CA, USA

**Keywords:** antibodies, anti-thymocyte globulin, human, N-glycolylneuraminic acid, sialic acids, Immunology

## Abstract

Humans have circulating antibodies against diverse glycans containing *N*-glycolylneuraminic acid (Neu5Gc) due to function-loss mutation of the *CMAH* gene. This xenogenic non-human carbohydrate is abundant in red meat, xenografts and biotherapeutics. Low levels of diet-derived Neu5Gc is also present on normal human endothelial cells, and together with anti-Neu5Gc antibodies could potentially mediate “xenosialitis” chronic-inflammation. Rabbit anti-human thymocyte globulin (ATG) is a drug containing polyclonal IgG glycoproteins commonly used as an immunosuppressant in human transplantation and autoimmune diseases. In type-1 diabetes patients, infusion of Neu5Gc-glycosylated ATG caused increased global anti-Neu5Gc response. Here, for the first time we explore changes in anti-Neu5Gc IgG repertoire following the immunization elicited by ATG, compared with the basal antibodies repertoire that reflect exposure to dietary-Neu5Gc. We used glycan microarrays with multiple Neu5Gc-glycans and controls to elucidate eventual differences in ATG-elicited repertoire, before/after ATG administration and track their kinetics (0, 1, 18 and 24 months). Response of all basal-pre-existing Neu5Gc-specific antibodies rapidly increased. This response peaked at one month post-ATG, with enhanced affinity, then resolved at 18–24 months. Induced-antibodies showed expanded diversity and *de-novo* recognition of different Neu5Gc-glycans, including endogenous glycolipids, that was further validated by affinity-purified anti-Neu5Gc antibodies from patients’ sera. These findings strongly suggest that ATG-induced anti-Neu5Gc IgGs represent a secondary exposure to this dietary carbohydrate-antigen in humans, with immune memory. Given their modified recognition patterns, ATG-evoked anti-Neu5Gc antibodies could potentially mediate biological effects different from pre-existing antibodies.

## INTRODUCTION

Humans develop a comprehensive immune response against Neu5Gc, a foreign dietary sialic acid form, with significant potential implications on chronic inflammation-mediated diseases such as cancer, atherosclerosis and xeno-transplantation [1–4]. Human anti-Neu5Gc antibodies behave differently from many other anti-carbohydrate antibodies [5]. These xeno-autoantibodies appear in infants coinciding with exposure to dietary Neu5Gc [6], are enhanced during disease [7, 8], class switched switch from IgM to IgG/IgA [9, 10]. Despite variable recognition profiles between human sera [9, 11], the levels can remain high for many years within the same individual [10]. Unlike other mammals, humans cannot hydroxylate cytidine 5’-monophosphate-*N*-acetylneuraminic acid (CMP-Neu5Ac) to synthesize Neu5Gc due to a specific gene loss-mutation. Instead, humans get exposed to Neu5Gc through dietary consumption of mammalian meat and dairy [1–4], and through biomedical exposure to Neu5Gc-glycosylated animal-derived tissues [2], biotherapeutics or bio-devices that are commonly used in the clinic [4, 12]. While humans cannot synthesize Neu5Gc, dietary-Neu5Gc can nevertheless be absorbed by human cells with subsequent low level expression on the surface of endothelial cells and some epithelial cells generating xeno-auto-antigens [1]. The combination of circulating anti-Neu5Gc antibodies with accumulated Neu5Gc on human cells leads to a unique situation of chronic inflammation defined as ‘xenosialitis’ [13, 14], that had been suggested to exacerbate various human diseases [3]. Experimental xenoxialitis had been demonstrated in mice [13, 14], yet in humans it may be even more complex and could likely be affected by changes in anti-Neu5Gc repertoire, for example by antibodies elicited through a non-dietary route. Therefore, deciphering the nature and repertoire of bioterapeutic-elicited anti-Neu5Gc antibodies could be critical for understanding their possible role in the immunopathogenesis of related chronic-inflammation mediated diseases. Neu5Gc and Neu5Ac, that differ by a single oxygen, are the two major sialic acids forms in mammals, which are ubiquitously expressed at the tip of diverse carbohydrate chains (glycans) on glycoproteins and glycolipids [1, 15–17]. It is challenging to measure anti-Neu5Gc antibodies, because they recognize multiple Neu5Gc-glycan epitopes, on a large collection of Neu5Gc-containing sugar chains. This variety results from Neu5Gc attachment to diverse underlying sugar-chains, with different linkages, glycan conjugation to protein/lipid-carriers and their diverse cell-surface density. To estimate overall anti-Neu5Gc response, a simple ELISA Inhibition Assay (EIA [7]) has been developed based on reactivity against multiple mouse Neu5Gc-containing glycoproteins, yet detailed information can only be achieved by measuring responses to distinct Neu5Gc-glycans with Neu5Ac-glycans counterparts serving as controls, facilitated by large scale analysis using printed arrays [9, 18, 19].

Rabbit anti-thymocyte globulin (ATG) is a polyclonal IgG preparation used for induction treatment of immunosuppression during various types of solid organ allografts, allogeneic stem cell transplants and autoimmune diseases [20]. These rabbit glycosylated antibodies carry determinants foreign to humans, including Neu5Gc-xenoantigens [4]. ATG therapy was administered to patients with new onset type-1 diabetes without additional immunosuppression in the START (Study of Thymoglobulin to ARrest Type 1 diabetes ) study, a phase II clinical trial (ClinicalTrials.gov #NCT00515099) that aimed to evaluate its effect on preservation of β-cell function [21, 22]. In these patients, ELISA-EIA analysis revealed a significant increase of both global anti-Neu5Gc IgM and IgG after ATG treatment [23]. Here, we use glycan microarray to profile the kinetics, specificity, affinity and repertoire diversity of anti-Neu5Gc IgGs following ATG treatment. The top seven EIA-responders were analyzed over a large panel of Neu5Gc-glycans and Neu5Ac-glycans on microarrays. This revealed that the drug-induced anti-Neu5Gc response is dramatically different from the basal response, with a strong induction of all pre-existing anti-Neu5Gc IgGs, as well as newly developed IgG repertoires against diverse Neu5Gc-glycans, with increased binding-affinity.

## RESULTS

To investigate carbohydrate immune recognition in the context of anti-Neu5Gc response, we took advantage of the availability of serum samples from the seven START study participants [21, 22] that had the highest titers of anti-Neu5Gc IgG one month following the four-days course of rabbit ATG treatment (Thymoglobulin-Genzyme; day 1: 0.5 mg/kg, days 2-4: 2 mg/kg; without additional immunosuppressive agents) [23], as determined by EIA [7] (Table 1). ATG targets multiple T-cell antigens, and its immunosuppressive activity is largely associated to peripheral T cell depletion, however it also affects other cell types [20, 24].

**Table 1:** Patients characteristics

	Treatment
	(n = 7)
Age in years	20 (7.8)
12–21 years	3 (43%)
22–35 years	4 (57%)
Men	4 (57%)
Ethnic origin	
White	6 (86%)
Non-white	1 (14%)
Body-mass index	22.4 (2.2)
Days since diagnosis	62.7 (24.4)
Baseline 2-h C-peptide area under the curve (pmol/ml)	0.764 (0.219)
Anti-Neu5Gc IgG (ng/μl)^*^	
Baseline	5.4 (2.3)
Month 1	212.4 (368.9)

^*^Measure by EIA assay [7].

**Figure 1:**
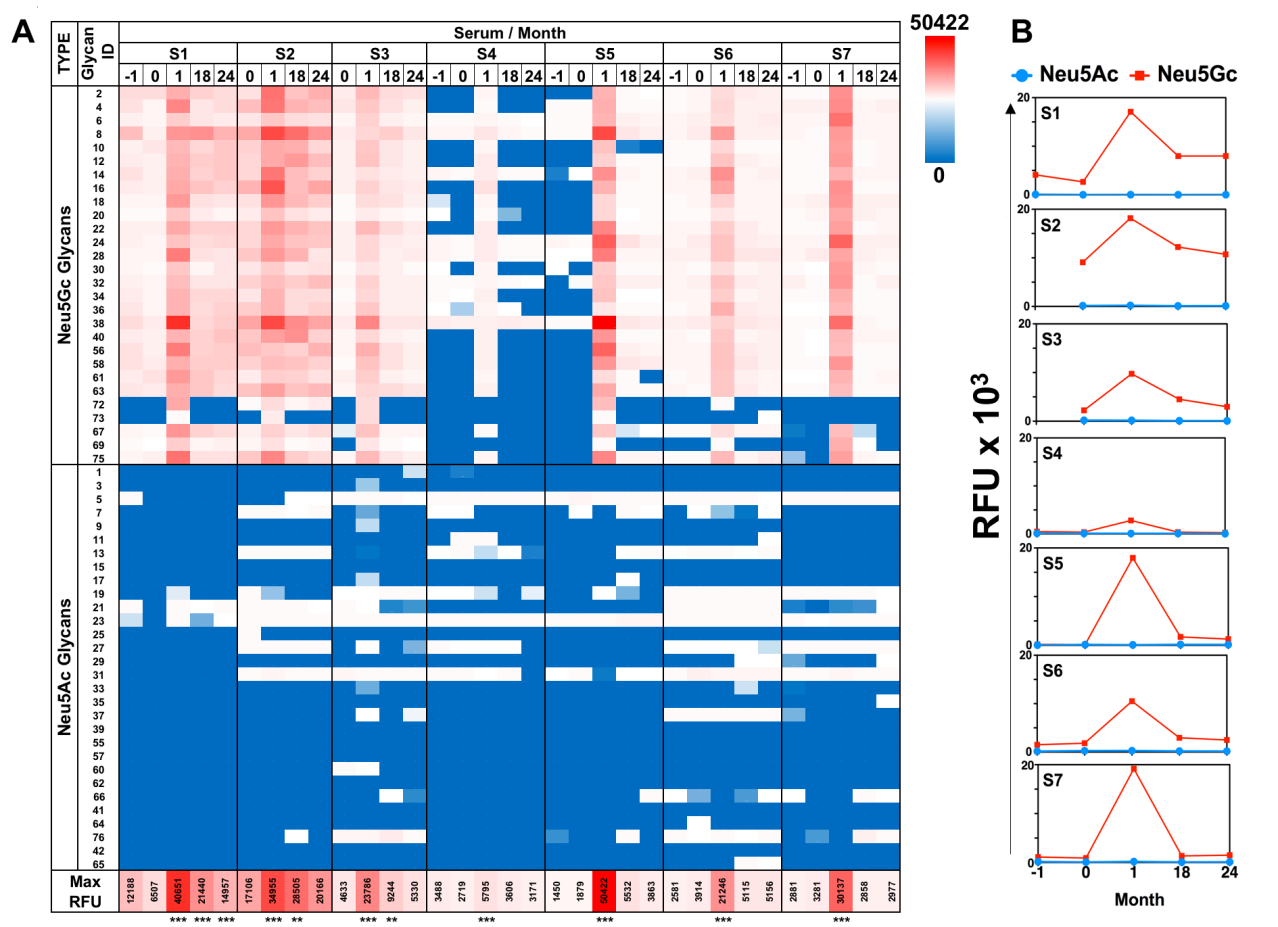
Diverse anti-Neu5Gc IgG response is induced after ATG treatment **A.** Sequential sera samples pre-/post-ATG therapy were tested at 1:100 dilution on sialoglycan microarrays, then detected by Cy3-anti-human IgG (40 ng/well). Relative fluorescence units (RFU) of all mono-sialylated Neu5Ac-glycans or Neu5Gc-glycans showed induction of highly specific anti-Neu5Gc response that peaked at one month post-ATG with some Neu5Gc-glycans responses sustained at 18-24 months post-ATG (Heatmap across all samples: red-white-blue represent maximum – 50^th^ percentile – minimum reactivity, respectively; minimum and maximum values are indicated in the figure; repeated measures One-Way ANOVA, ^***^ *P* < 0.0001 significant between M0 and M1; detailed analysis described in Supplementary Figure 1A). Glycan structures are detailed in Supplementary Table 1. **B.** Average RFU of all mono-sialylated Neu5Ac-glycans (blue line) or Neu5Gc-glycans (red line) showed induction of highly specific anti-Neu5Gc response peaking one month post-ATG.

**Figure 2:**
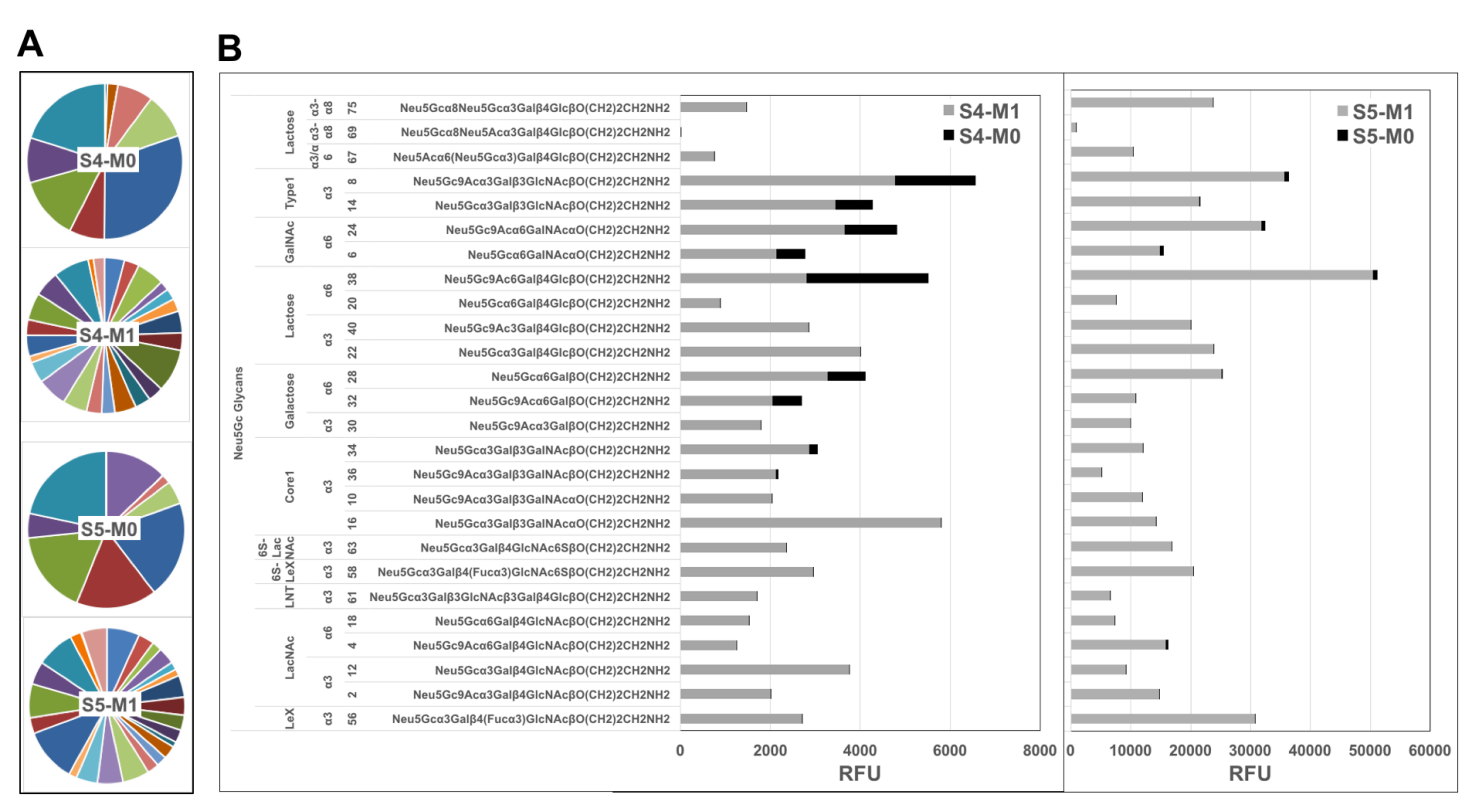
Diverse de novo anti-Neu5Gc IgGs repertoire post-ATG **A.** Pie charts of anti-Neu5Gc IgG microarray recognition patterns against each Neu5Gc-glycan in S4 and S5 pre-ATG (M0) and post-ATG (M1) exemplifies increased diversity (each pie faction represents a different Neu5Gc-glycan, demonstrating increased number of Neu5Gc-glycans that are being recognized at M1 compared with M0 in each sera, reflected by increased number of pie fractions in M1). **B.** S4 and S5 RFU divided to common underlying core-glycan-structures demonstrate *de novo* recognition of additional Neu5Gc-glycans with strongly increased intensities.

**Figure 3:**
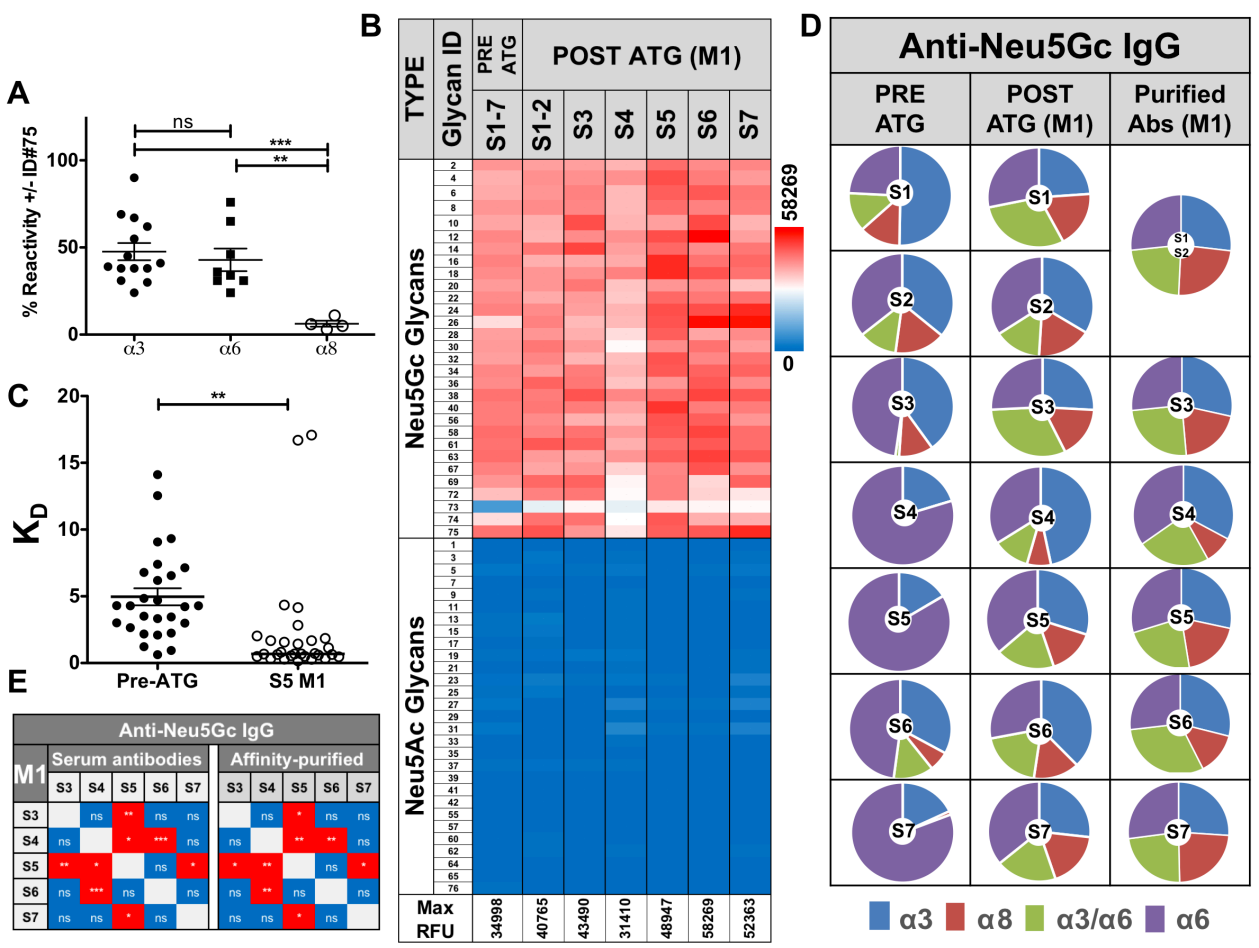
Characterization of affinity-purified anti-Neu5Gc IgGs pre- and post-ATG **A.** Anti-Neu5Gc antibodies were affinity-purified from pooled pre-ATG or S5-M1 sera then 1 μg/well each analyzed by sialoglycan microarrays, in the presence or absence of 0.5 mM of competing glycan ID#75 (Neu5Gcα8Neu5Gcα3Galβ4GlcβO-Linker), followed by detection with Cy3-anti-human IgG (40 ng/well). Reactivity against glycolipid-glycans (α8) is greatly inhibited compared to others (α3/6) (One-way AVONA, Bonferroni post-test, *p* = 0.0012). **B.** Affinity-purified antibodies tested at 1 μg/well followed by Cy3-anti-human IgG confirmed high Neu5Gc-specificity (Heatmap across all samples). **C.** To evaluate affinities of purified anti-Neu5Gc antibodies from pre-ATG *versus* S5-M1 sera, IgGs reactivity was tested at 14 quartile-serial dilutions and K_D_-values/glycan calculated, demonstrating increased affinities post-ATG (medians; non-linear fit with one-site specific binding; Table S2). **D.** Pie charts of anti-Neu5Gc-IgG divided by Sia-linkages reveals increased diversity pre-/post-ATG, that is maintained after affinity-purification. Charts represent average response against Neu5Gc-glycans with Siaα2–3 linkage (α3), Siaα2–6 linkage (α6), glycolipid-type di/tri-sialylated with Siaα2–8 linkage (α8), or the branched di-Sia (α3/6). **E.** Similar correlation of anti-Neu5Gc IgG at M1-post-ATG before/after affinity-purification between patients (Pearson, Two-tailed, 95% CI), supporting efficient affinity-purification.

For detailed profiling of anti-Neu5Gc immune responses we used a unique sialoglycan microarray with various Neu5Gc-glycans and control Neu5Ac-glycans, each covalently attached to epoxy-activated glass slides [7]. We focused on IgG response that had been suggested to mediate chronic inflammation diseases, and for this purpose used anti-human IgG specific secondary antibody that had been validated to lack cross reactivity with other human Ig isotypes (i.e. IgM, IgA). To characterize the kinetics of response to these diverse glycans we analyzed patients’ serum samples obtained before-ATG [screening visit (-1), day of treatment (0; before administration)], and samples taken after treatment (at 1, 18 and 24 months). In all patients, there was a clear induction of highly-specific anti-Neu5Gc IgG response, with minimal/no recognition of control Neu5Ac-glycans (Figure 1), confirming previous EIA analysis [23]. Anti-Neu5Gc responses were increased vigorously, peaking at one month post-ATG infusion, then resolved at 18-24 months (Figure 1, Supplementary Figure 1). This exemplifies a long-term effect despite a short initial antigenic challenge, supporting previous studies in patients shortly exposed to animal-derived tissues [10]. At one month, all pre-ATG-M0 antibodies were elevated, and in addition, antibodies to new epitopes were detected in sera of most subjects, showing that ATG-treatment increased the repertoire diversity by eliciting *de novo* recognition of individual Neu5Gc-glycans (Figure 1A, Supplementary Figure 1B; for example, glycan ID #73 in S1-S3, and glycan IDs #72, #73, #69, #67, #75 in S5). The most prominent examples of this were observed for S4/S5-microarrays (Figure 2A-2B). These newly generated anti-Neu5Gc IgGs were not detected prior to ATG treatment at month 0, and recognized several mono-sialylated α2-3/6-linked glycans and the glycosphingolipid oligosaccharide α2-8-linked Neu5Gc-di-sialylated glycan (ID#75: Neu5Gcα8Neu5Gcα3Galβ4GlcβO-Linker; Figure 2B). Importantly, this glycolipid-glycan recognition was highly specific with low cross-reactivity to other Neu5Gc-glycans, as demonstrated by differential reactivity-inhibition with glycan ID#75 (Figure 3A). Glycolipid recognition was unexpected since ATG-IgGs are glycoproteins containing *N*-glycans that lack Siaα2-8, as determined by mass-spectrometry [4]. Lectin analysis showed ATG-IgGs carry both Siaα2-3 and Siaα2-6, with HPLC-quantified Neu5Gc (3.1 ± 0.05 pmole/μg ATG) at ~7-fold over Neu5Ac (Supplementary Figure 2). At one month, the average total anti-Neu5Gc IgG response was dramatically enhanced in S4 by 7-fold and in S5 by 122-fold (S4: 343 ± 131 to 2495 ± 251; S5: 142 ± 52 to 17338 ± 2157; RFU mean ± SE) (Figure 2B). In both S4 and S5, *de novo* induced anti-Neu5Gc IgGs peaked at one month post-ATG (M1), but only in S5 remained in circulation through 18-24 months (Figure 1), suggesting differences in the quality of the humoral response elicited by ATG in different individuals, and also that introduction of these animal-derived Neu5Gc-containing glycoproteins sometimes result in a long-lasting antibody exposure to new Neu5Gc-epitopes.

To directly compare the quality of pre-existing *versus* ATG-induced anti-Neu5Gc antibodies from these patients’ sera, anti-Neu5Gc IgGs were affinity-purified. Sera were loaded on two sequential columns with the first containing Neu5Ac-glycoproteins, and the second containing Neu5Gc-glycoproteins, from which anti-Neu5Gc antibodies were eluted with free Neu5Gc [9]. Due to expected low quantities of anti-Neu5Gc antibodies in pre-ATG sera (Figure 1), serum from all seven patients collected before ATG-treatment were pooled together for affinity-purification, yielding 3.2 μg/ml pooled sera (total 10 ml). At the one month time point, anti-Neu5Gc antibodies were affinity-purified from individual sera, resulting in 30.8 ± 13.7 μg/ml sera (mean ± SE), demonstrating at least 10-14-fold more antibodies in the elicited response. These yields likely represent most anti-Neu5Gc reactivity due to minimal residual-reactivity in the column flow-through after purification (data not shown). Glycan microarray analysis of these purified anti-Neu5Gc antibodies confirmed their high specificity towards all Neu5Gc-glycans, and not towards Neu5Ac-glycans (Figure 3B). These elevated antibody quantity yields at M1 compared to pooled pre-ATG levels were also associated with a major enhancement in reactivity, as exemplified in S4/S5 (Figure 2B).

To test whether the increased reactivity against various Neu5Gc-epitopes was also associated with enhanced binding strength, we evaluated the affinity equilibrium constant K_D_. Due to limited sample availability, K_D_ could not be determined from kinetic measurements (the ratio of the rate constants k_d_/k_a_). Instead it was estimated through glycan microarray analysis, by fitting a plot of response at equilibrium against a wide range of purified anti-Neu5Gc antibody concentrations, with a constant concentration of each of the printed Neu5Gc-glycans (at 100 μM). This array-titration analysis of purified-antibodies suggest a ~7-fold increased IgG-affinity at S5-M1 *versus* pre-ATG (0.2-22.1-fold K_D_ increase against individual Neu5Gc-glycans; Figure 3C, Supplementary Table 2). To further evaluate the efficiency of affinity-purification to obtain the authentic pool of induced antibodies from each serum, we analyzed profiles of average Neu5Gc-glycans responses against specific Sia-linkages (Siaα2-3/6/8; Figure 3D). This confirmed the expanded repertoire diversity in elicited anti-Neu5Gc antibodies post-ATG. Furthermore, the affinity-purified antibodies maintained their unique serum-repertoire patterns, and their differences from other samples (Figure 3D). Likewise, correlation patterns between sera before/after purification remained the same (Figure 3E). Altogether, these data provide direct evidence that the short treatment with Neu5Gc-glycosylated foreign ATG not only causes a quantitative induction, but also a qualitative enhancement of highly specific anti-Neu5Gc IgGs, with improved affinities. Importantly, this highly-specific and diverse induced response is not limited to Neu5Gc-antigens on ATG-protein-carriers, but can also recognize Neu5Gc-glycolipids, implying a recall response with activation of initially-quiescent memory.

## DISCUSSION

Antibody secretion is initiated by activated-B cells that differentiate into short- or long-lived plasma cells. To diversify the repertoire of antigen-specific cells and optimize their affinity, activated-B cells transiently repress plasma cell (PC) differentiation followed by class-switch recombination (e.g. to IgG), or that they enter the germinal center (GC) for somatic hypermutation and class-switch recombination to yield high-affinity clonal variants that differentiate into either quiescent memory-B cells or plasma cells [25]. Some GC-plasma cells turn into long-lived plasma cells (LLPCs) in the bone marrow, where they do not proliferate, but act as long-term antibody factories [25, 26].

The enhanced and diverse anti-Neu5Gc immune response following Neu5Gc-ATG treatment is thus likely mediated by memory-B cells, that could include both quiescent memory-B cells and LLPCs. At one month, the dramatic increase in pre-existing anti-Neu5Gc IgGs is in contrast to the overall transient B cell depletion observed in these patients [21]. Furthermore, the increase in anti-Neu5Gc IgGs repertoire-breadth, with *de novo* recognition patterns, supports the anamnestic involvement of quiescent memory-B cells that become activated upon re-exposure to Neu5Gc-glycosylated-ATG. This is further corroborated by the recognition of Neu5Gc-glycolipid glycans not expected to appear on ATG-glycoproteins.

This study was limited to antibody repertoires in patients’ serum samples, lacking accessibility to blood cells in ‘real time’, yet the elaborated results strongly substantiate the existence of memory responses to the Neu5Gc-dietary carbohydrate antigen. Circulating anti-Neu5Gc antibodies may contribute to exacerbation of chronic inflammation-mediated diseases [8, 27], including in cancer and vascular lesions [14, 28–31], but are also potential therapeutics or biomarkers [1, 3, 32]. Our findings provide the first description of the drug-elicited anti-Neu5Gc IgGs with different repertoires and affinities. These may contribute to as yet unidentified pathologies in patients exposed to high levels of such elicited-antibodies for long periods after stimulation with animal-derived molecules or tissues [4, 12]. Drug-induced anti-Neu5Gc antibodies could potentially recognize integrated dietary-Neu5Gc on endothelial/epithelial cells [1, 3], and interfere with their proper functions. In this respect, our findings suggest that further investigation of induced anti-Neu5Gc antibodies is warranted, especially those being elicited in patients challenged with animal-derived drugs, grafts, tissues or bioprosthetic devices.

## MATERIALS AND METHODS

### Human sera samples

Seven patients’ sera were obtained from the START study (Gitelman et al., 2013) (Table 1) and used in accordance with the Helsinki declaration and Tel Aviv University Institutional Review Board.

### Antibodies

Neu5Gc IgY (Biolegend), biotinylated-SNA and biotinylated-MAL-II (Vector Labs), HRP-donkey-anti-chicken IgY, HRP-Streptavidin and Cy3-goat-anti-human-IgG (H+L) (Jackson ImmunoResearch).

### Affinity purification of anti-Neu5Gc antibodies from human sera

Antibodies were purified as previously described [9]. The sera of all seven patients at M(-1) and M0 were pooled resulting in 32.3 μg of the pooled 10 ml. Yields of sera at the peak response (M1) were 73.8 μg/ml S1-S2 (221.4 μg of 3 ml pooled S1 and S2), 73.3 μg/ml S3 (183.1 μg of 2.5 ml), 6.7 μg/ml S4 (13.4 μg of 2 ml), 18.4 μg/ml S5 (36.8 μg of 2 ml), 7.5 μg/ml S6 (1.87 μg of 0.25 ml), and 4.9 μg/ml S7 (9.8 μg of 2 ml).

### Sialoglycan microarray fabrication

Arrays were fabricated with NanoPrint LM-60 Microarray Printer (Arrayit) on epoxide-derivatized slides (Corning 40044), at 70% humidity, with four SMP3 pins (Arrayit) at sixteen 20×20 sub-arrays on each slide (Version 2.0). Each glycoconjugate printed in 100 μM of optimized print buffer (300 mM phosphate buffer, pH 8.4). Next, slides were packed, vacuum-sealed and stored at room temperature (RT) until used.

### Sialoglycan microarray binding assay

Slides were developed and analyzed as previously described [7] with some modifications. Slides were rehydrated with ddH_2_O and incubated for 30 min in a staining dish with 50 °C pre-warmed ethanolamine (0.05 M) in Tris-HCl (0.1 M, pH 9.0) to block the remaining reactive epoxy groups on the slide surface, then washed with 50 °C pre-warmed ddH_2_O. Slides were centrifuged at 200 × *g* for three min then fitted with ProPlate™ Multi-Array 16-well slide module (Invitrogen) to divide into the sub-arrays (blocks). Slides were washed with PBST (0.1 % Tween 20), aspirated and blocked with 200 μl/sub-array of blocking buffer (PBS/OVA, 1% w/v ovalbumin, in PBS, pH 7.3) for 1 hour at RT with gentle shaking. Next, the blocking solution was aspirated and 100 μl/block of 1:100 diluted sera or purified anti-Neu5Gc antibodies in 10 ng/μl diluted in PBS/OVA were incubated with gentle shaking for 2 hours at RT. Slides were washed three times with PBST, then with PBS for 2 min. Bound antibodies were detected by incubating with secondary detection diluted in PBS, 200 μl/block at RT for 1 hour, Cy3-anti human IgG 1.2 μg/ml (Jackson Immunoresearch). Slides were washed three times with PBST then with PBS for 10 min followed by removal from ProPlate™ Multi-Array slide module and immediately dipping in a staining dish with ddH_2_O for 10 min with shaking, then centrifuged at 200 × *g* for 3 min and scanned immediately.

### Array slide processing

Processed slides were scanned and analyzed as described at 10 μm resolution with a Genepix 4000B microarray scanner (Molecular Devices) at 350 gain. Image analysis was carried out with Genepix Pro 6.0 analysis software (Molecular Devices). Spots were defined as circular features with a variable radius using with local background subtraction.

### Affinity equilibrium constant KD calculation by microarray

Slides were developed as described above with serial quarterly dilutions of purified S5-M1 or purified pooled Pre-ATG antibodies at 10 ng/μl − 1.49 × 10^-7^ ng/μl (66.6 nM − 9.93 × 10^-7^ nM) in PBS/OVA blocking buffer. K_D_ was calculated by fitting a plot of response at equilibrium against a wide range of purified anti-Neu5Gc antibody concentrations (12-13 dilutions, from 9.93 × 10^-7^ nM up to 4.16 nM or 16.6 nM, in S5-M1 and pooled Pre-ATG, respectively; non-linear fit with one-site specific binding, GraphPad Prism 6.0).

### Selective inhibition with glycan ID #75 by microarray

Slides were developed as described with the following modifications. Purified 10 ng/μl of S5-M1 were pre-incubated in the presence or absence of 0.5 mM glycan ID #75 in PBS/OVA blocking buffer for 2 hours on ice prior to loading on the slide for development.

### Sialic acid detection by ELISA

Sialic acids were detected on ATG by ELISA as previously described [9]. ATG was coated in duplicates at 1μg/well in 50 mM sodium carbonate-bicarbonate buffer, pH 9.5 onto 96-well microtiter plates (Costar, Corning) and plates were incubated overnight at 4 °C. Wells were blocked for 1 hour at room temperature with blocking buffer (PBS pH 7.4, 1% ovalbumin), then aspirated and incubated with diluted primary antibody 100 μl/well in the same blocking buffer for two hours at room temperature (chicken anti-Neu5Gc IgY at 1:7000, biotinylated SNA or MAL-II at 1 μg/ml). The plates were washed three times with PBST (PBS pH 7.4, 0.1% Tween-20) and subsequently incubated for 1 hour at RT with HRP-conjugated secondary antibody in PBS (HRP-donkey-anti-chicken IgY 0.26 μg/ml and HRP-streptavidin 0.09 μg/ml, respectively). After washing three times with PBST, wells were developed with 140 μl of *O*-phenylenediamine in 100 mM citrate-PO_4_ buffer, pH 5.5, and the reaction stopped with 40 μl of H_2_SO_4_ (4 M). Absorbance was measured at 490 nm on SpectraMax M3 (Molecular Devices). Specific binding was defined by subtracting the background readings obtained with the secondary antibody only on coated wells.

### Sialic acid analysis by DMB-HPLC

Sia content in ATG was analyzed as previously described [33]. Sias were released from glycoconjugates by acid hydrolysis with 0.1 M of H_2_SO_4_ for 1.5 hours at 80 °C, then neutralized with 0.1 M of NaOH. Free Sias were then derivatized with 1, 2-diamino-4, 5-methylenedioxybenzene (DMB; Sigma) for 2.5 hours at 50 °C, separated by Microcon-10 centrifugal filters and analyzed by fluorescence detection on reverse-phase high pressure liquid chromatography (DMB-HPLC; Hitachi HPLC Chromaster). HPLC run was on C18 column (Phenomenex C18 Gemini 250 × 4.6 mm) at 24 °C in running buffer [84.5% ddH_2_O, 8.5% acetonitrile, 7% methanol (Merck)] for 60 minutes (min) at a flow rate of 0.9 ml/min. Quantification of Sias was done in comparison with known quantities of DMB-derivatized Neu5Ac.

### Statistical analysis

Statistical analyses were performed using GraphPad Prism 5.0 or 6.0, and described in context in the figure legends. P-value less than 0.05 was considered statistically significant.

## SUPPLEMENTARY MATERIALS FIGURES AND TABLES




