# Supplementary material for: Glycan microarray reveal induced IgGs repertoire shift against a dietary carbohydrate in response to rabbit anti-human thymocyte therapy
Source: Oncotarget. 2017 Dec 11;8(68):112236–44. doi: 10.18632/oncotarget.23096 (PMC5762506; doi:10.18632/oncotarget.23096)
Supplement: Supplementary file 1 [file oncotarget-08-112236-s001.pdf]

Glycan microarray reveal induced IgGs repertoire shift against a dietary carbohydrate in response to rabbit anti-human thymocyte therapy

SUPPLEMENTARY MATERIALS

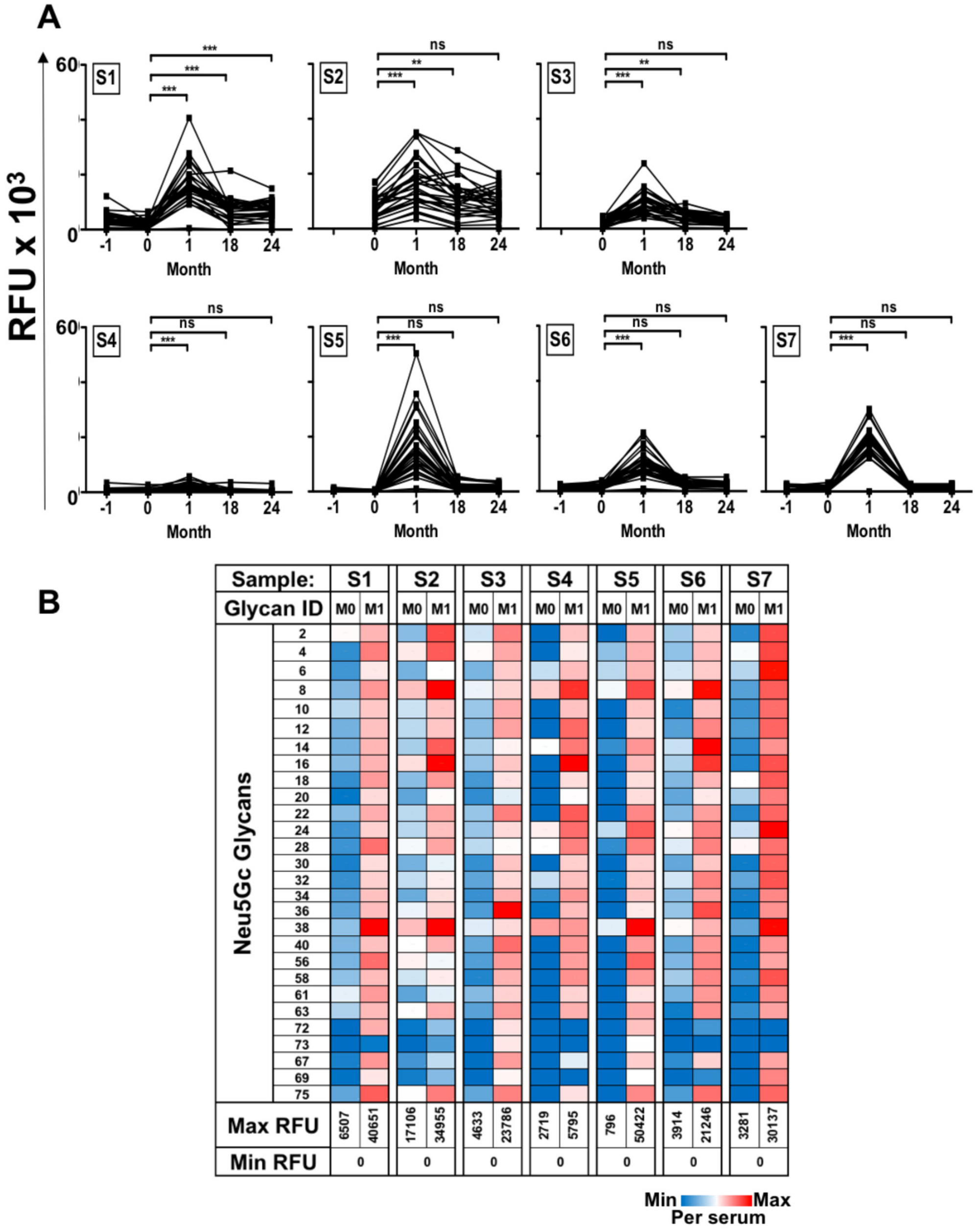

Supplementary Figure 1: Diverse anti-Neu5Gc IgG response is induced after ATG treatment. Related to Figure 1. (**A**) Sequential patients' serum samples pre-/post-ATG therapy were tested at 1:100 dilution on sialoglycan microarrays, detected by Cy3-anti-human IgG (40 ng/well) (repeated measures One-Way ANOVA, *** P<0.0001, with Bonferroni's Multiple Comparison Test). (**B**) Serum anti-Neu5Gc IgG repertoire measured against Neu5Gc-glycans pre-ATG (M0) and one month post-ATG (M1) revealed newly-derived antibodies (Heatmap per serum: red-white-blue represent maximum-50th percentile-minimum reactivity, respectively).

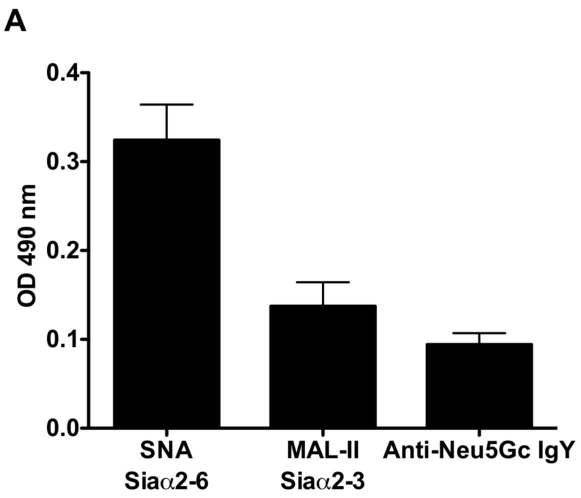

Supplementary Figure 2: Sialic acids on rabbit ATG. Related to Figure 3. (**A**) Sialic acids on ATG were demonstrated by ELISA using SNA lectin for Sia2–6-linked, MAL-II lectin for Siaα2–3-linked and chicken anti-Neu5Gc IgY for Neu5Gc detection. (**B**) Neu5Gc and Neu5Ac on ATG were quantified by DMB-HPLC.

For Supplementary Tables see in Supplementary Files.
